# Relationship Between Marijuana Use and Hospitalization for Acute Coronary Syndrome

**DOI:** 10.7759/cureus.23317

**Published:** 2022-03-19

**Authors:** Niraj Karki, Binita Sapkota, Swosti R Magar, Ameen Muhammad, Bishow M Paudel, Peter Chernek, Maryam Afshar, Manoj Bhandari, Jonathan N Bella

**Affiliations:** 1 Internal Medicine, Catholic Health Initiatives (CHI) St. Vincent, Little Rock, USA; 2 Rheumatology/Internal Medicine, University of Arkansas for Medical Sciences, Little Rock, USA; 3 Internal Medicine/Rheumatology, University of Arkansas for Medical Sciences, Little Rock, USA; 4 Internal Medicine, BronxCare Health System, Bronx, USA; 5 Internal Medicine, University of Iowa Hospitals and Clinics, Iowa City, USA; 6 Internal Medicine/Cardiology, BronxCare Health System, Bronx, USA; 7 Cardiology, Cape Fear Valley Health System, Fayetteville, USA

**Keywords:** marijuana use and hospitalization, cardiovascular effects, marijuana, acute coronary syndrome, urine toxicology

## Abstract

Background: Recreational marijuana use is rising, especially among young adults. The cardiovascular (CVD) effect of marijuana remains mostly unknown.

Methods: This is a retrospective study of 14,490 patients admitted to our hospital between 2012 and 2014 who had urine toxicology done for various reasons. Patients with a primary diagnosis of acute coronary syndrome (ACS) were queried in both the marijuana-positive group (n = 59) and the marijuana-negative group (n = 195). The risks of having ACS were compared in both groups.

Results: There was no difference in the risk of having ACS between the two groups in the population < 54 years of age (OR: 0.90, 95% CI: 0.67-1.20, p = 0.48). However, there was a significant difference in the risk of having ACS in the 18-36 age group (OR: 2.84, 95% CI: 1.14-7.07, p = 0.01). Multivariate analysis performed to adjust for the potential confounding effects of smoking and cocaine use showed that marijuana use (OR: 0.93, 95% CI: 0.68-1.25, p = 0.65) did not increase the likelihood of ACS for patients ≤ 54 years or for those in the 37-54 age group (OR: 1.11, 95% CI: 0.79-1.53, p = 0.50). However, among the 18-36 age bracket, marijuana use was independently associated with a higher risk of ACS (OR: 5.24, 95% CI: 1.84-16.93, p = 0.002).

Conclusion: In younger patients (age 18-36 years), marijuana use is independently associated with a five-fold higher risk of ACS.

## Introduction

Marijuana is one of the most commonly consumed recreational drugs in the United States (US) [1]. The use of marijuana has doubled since 2002 as demonstrated in a study on an in-patient population with an increasing trend toward use in the older and sick population [2]. Marijuana has been legalized for medical use in 33 states and Washington, DC, and for recreational use in 11 states [3]. There have been several case reports of myocardial infarction (MI) caused by marijuana consumption [4-8]. Marijuana use has been known to increase the mortality rate in patients who had MI by three-fold with a graded increase in mortality with frequent use [9]. In the Determinants of Myocardial Infarction Onset Study, it was found that the risks of MI rapidly increased in the first 60 minutes following marijuana use [10]. A recent study on a large nationwide in-patient sample concluded that marijuana use increased the likelihood of acute ischemic stroke hospitalization by 17% [11]. However, there have not been any large population-based studies to determine the effects of marijuana use on the heart.

The major pharmacologically active component of marijuana is delta-9-tetrahydrocannabinol (THC), which acts on cannabinoid receptor 1 (CB1) and cannabinoid receptor 2 (CB2) receptors. The CB1 receptor is pro-atherogenic, while the CB2 receptor is anti-atherogenic [12]. The acute effect of marijuana on the cardiovascular system are tachycardia, increased cardiac work, hypertension, and vasodilatation. The long-term effects include an increased chance of angina, MI, cardiomyopathy, and cardiac death [13]. Due to the scarce evidence on the cardiovascular effects of marijuana, we performed a retrospective analysis of patients admitted to our hospital to assess the in-patient risk of acute coronary syndrome (ACS) in marijuana users versus non-users.

## Materials and methods

Study design

This was a retrospective cohort study conducted at a community hospital in Bronx, New York. The study cohort was obtained from hospital admissions between January 2012 and December 2014, if they had urine toxicology done for any reason. The cohort was further divided into marijuana-positive and marijuana-negative groups based on urine toxicology findings. The risks of having ACS were compared between the marijuana-positive and marijuana-negative groups.

Data source and inclusion criteria

Data were collected from electronic medical records by running queries and individual chart reviews. Hospitalized patients between 18 and 54 years were included in the study if they were found to have urine toxicology done for any reason. Patients above 54 years were excluded from the study to avoid the confounding effects of age and other cardiovascular risk factors seen in the older population on ischemic heart disease. We used the international code of diseases (ICD)-10 code to query the diagnosis of unstable angina, non-ST-elevation myocardial infarction (NSTEMI), and ST-elevation infarction (STEMI) and grouped them as ACS. The risks of ACS were compared in marijuana users versus non-users.

Variables

The demographic variables compared between the two groups were age, sex (male or female), and race (African American, Hispanics, Caucasian, or others). Other variables compared between the two groups were body mass index (BMI), personal history of coronary artery disease (CAD), hypertension, dyslipidemia, diabetes, peripheral vascular disease, and family history of CAD.

Smoking status was recorded based on the documented history, and cocaine use was charted based upon the urine toxicology findings. The total length of hospital stay and in-hospital deaths were also calculated for both groups and compared. If the study cohort had a nuclear stress test or left heart cardiac catheterization done, they were also charted, and results were documented as positive or negative.

Statistical analysis

Baseline characteristics of the study population were defined using descriptive statistics (mean and standard deviation for continuous variables; numbers and percentages for categorical variables). Multivariable logistic regression calculating the odd ratios of ACS for those with marijuana use compared to patients without marijuana use was done. The Odds ratio was also calculated to show the chances of having ACS by age group, and a 95% confidence Interval was calculated. Any p-value less than 0.05 was considered significant. All statistics reported were performed using RStudio open-source software (Boston, MA).

## Results

Subject characteristics

**Figure 1:**
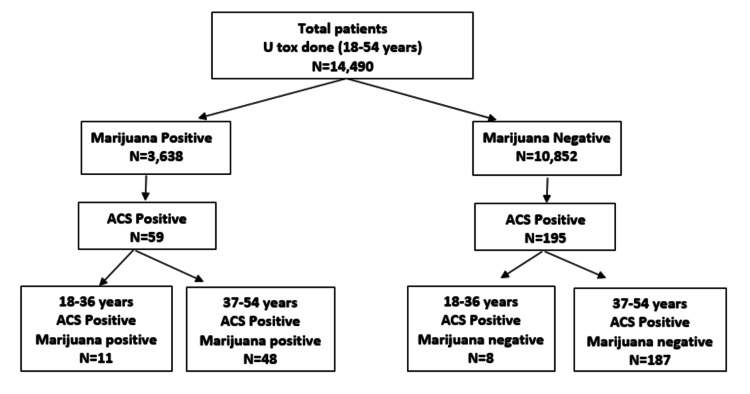
Distribution of the study population U tox: Urine toxicology; ACS: Acute coronary syndrome.

Between 2012 and 2014, a total of 14,490 patients who were 54 years old and under were admitted to our hospital who had urine toxicology done for various reasons. Among them, 3638 patients had positive urine toxicology results for marijuana, and 10,852 had negative urine toxicology results; 59 out of 3638 patients were found to be having ACS, while 195 out of 10,852 patients had ACS (Figure 1).

The mean age was 45.3 ± 7.8 years in the marijuana-positive group with ACS, while the mean age was 47.2 ± 5.4 years in the marijuana-negative group with ACS. Sixty-one percent (61%) of marijuana users who had ACS were male, 44% were African American, 19% were Hispanic, 44% were dyslipidemic, 76% were hypertensive, and 44% were diabetic (Table 1). Demographics and CVD risk factors were similar in both groups with p > 0.05 except for smoking and cocaine use. Thirty-nine percent (39%) of marijuana users were smokers, while only 22% of non-users were smokers (p = 0.015). Similarly, 27% of marijuana users with ACS had urine toxicology positive for cocaine, while only 13% of marijuana non-users who had ACS were cocaine users (p = 0.02).

**Table 1:** Descriptive results of selected clinical variables by marijuana use status (positive versus negative) in patients having acute coronary syndrome (ACS) ^i^Chi-square test ^ii^Fisher's exact test ^iii^Wilcoxon rank-sum test SD: Standard deviation; CAD: Coronary artery disease; PVD: Peripheral vascular disease.

Total (N = 254)	Marijuana Use (N = 59)	Non-marijuana Use (N = 195)	p-value
Age (Mean) SD	45.3 (±7.8)	47.2 (±5.4)	0.2886^iii^
Gender			0.4731^i^
Female	23 (39%)	64 (33%)	
Male	36 (61%)	131 (67%)	
Ethnicity			0.2895^ii^
African American	26 (44%)	71 (37%)	
Hispanic	11 (19%)	49 (25%)	
Caucasian	0 (0%)	8 (4%)	
Other	22 (37%)	67 (34%)	
Obesity			0.05828^i^
Yes	20 (34%)	99 (51%)	
No	38 (66%)	95 (49%)	
Family history of CAD			0.0900^i^
Yes	17 (29%)	42 (22%)	
No	41 (71%)	153 (78%)	
Dyslipidemia			0.6850^i^
Yes	26 (44%)	42 (22%)	
No	33 (56%)	117 (96%)	
Smoker			0.01500^i^
Yes	23 (39%)	43 (22%)	
No	36 (61%)	152 (78%)	
Diabetes			0.9011^i^
Yes	26 (44%)	82 (42%)	
No	33 (56%)	113 (58%)	
Hypertension			0.9003^i^
Yes	45 (76%)	145 (74%)	
No	14 (24%)	50 (26%)	
PVD			1.0000^ii^
Yes	1 (2%)	6 (3%)	
No	58 (98%)	189 (97%)	
CAD			0.7819^i^
Yes	23 (39%)	70 (56%)	
No	36 (61%)	125 (44%)	
Cocaine			0.0216^i^
Yes	16 (27%)	26 (13%)	
No	43 (73%)	169 (87%)	

Risks of ACS

**Figure 2:**
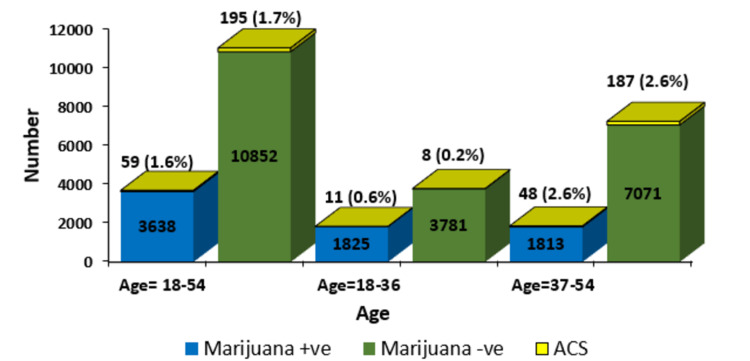
Acute coronary syndrome incidence according to age groups in marijuana-positive and marijuana-negative patients +ve: Positive; -ve: Negative; ACS: Acute coronary syndrome.

The chances of having ACS between the two groups were not different (OR: 0.90, 95% CI: 0.67-1.20, p = 0.48) in the study cohort (Table 2). We performed a subgroup analysis by splitting the total population (age: 18-54 years) into two age groups of 18-36 years and 37-54 years (Figure 2). We found a significant difference in the risk of having ACS in the 18-36 age group (OR: 2.84, 95% CI: 1.14- 7.07, p = 0.01), while this significant finding was not seen in the 37-54 age group (OR: 1.00, 95% CI: 0.73-1.36, p = 0.99).

**Table 2:** Odds ratio of having acute coronary syndrome (ACS) for marijuana users compared to non-users within three different age groups

Age Group	Odds Ratio	p-value	95% Confidence Interval
18-54 years (n = 14,490)	0.9025	0.4861	0.676-1.204
18-36 years (n = 5,606)	2.8486	0.0182	1.148-7.070
37-54 years (n = 8,848)	1.0011	0.9944	0.732-1.369

Both tobacco use and cocaine use were found to be significantly higher in marijuana users compared to non-users. We performed a multivariate analysis to adjust for the potential confounding effects of smoking and cocaine use. Overall, marijuana use (OR: 0.93, 95% CI: 0.68-1.25, p = 0.65) did not increase the likelihood of ACS for patients in the 18-54-year age group (Table 3). However, in the subgroup analysis, marijuana use showed a significant increase in the risks of having ACS within the 18-36 age bracket (OR: 5.24, 95% CI: 1.84-16.93, p = 0.002) but not in the 37-54 age group (OR: 1.11, 95% CI: 0.79-1.53, p = 0.50).

**Table 3:** Logistic regression model of marijuana use on ACS adjusting for smoking and cocaine use within the total population (18-54 years) and two specific age groups (18-36 years and 37-54 years) ACS: Acute coronary syndrome.

	Odds Ratio	p-value	95% Confidence Interval
Total sample (n = 14,490)			
Marijuana positive	0.9349	0.657	0.6889-1.2502
Smoker	0.7643	0.074	0.5650-1.0207
Cocaine user	1.0151	0.932	0.7080-1.4220
Age group (18-36 years) (n = 5,606)			
Marijuana positive	5.244	0.0027	1.847-16.936
Smoker	2.603	0.20181	0.517-10.969
Cocaine user	1.506	0.52997	0.339-4.768
Marijuana smoking		0.03024	
Age group (37-54 years) (n = 8,884)			
Marijuana positive	1.117	0.5089	0.797-1.536
Smoker	0.644	0.0043	0.473-0.866
Cocaine user	0.757	0.1296	0.522-1.073

Types of ACS

In the marijuana-positive group with ACS, 32.2% had unstable angina, 38.9% had NSTEMI, and 28.8% had STEMI. In the marijuana-negative group with ACS, 38.9% had unstable angina, 43.5% had NSTEMI, and 17.4% had STEMI (Table 4). There was a significant difference in comparing patients with STEMI between the marijuana-positive group and the marijuana-negative group with p = 0.05. In both groups, a higher number of ACS patients had MI compared to unstable angina; however, this difference was not statistically significant (Table 5).

**Table 4:** Breakdown of ACS into unstable angina, NSTEMI, and STEMI in both marijuana-positive and marijuana-negative groups ^i^p-values calculated using z-scores for comparison of population proportions. ACS: Acute coronary syndrome; NSTEMI: Non-ST-segment elevation myocardial infarction; STEMI: ST-segment elevation myocardial infarction.

Total (N = 254)	Marijuana positive with ACS (N = 59)	Marijuana negative with ACS (N = 195)	p-value
Unstable angina (n = 91)	19 (32.2%)	76 (38.9%)	p = 0.3523^i^
NSTEMI (n = 108)	23 (38.9%)	85 (43.5%)	p = 0.5287^i^
STEMI (n = 51)	17 (28.8%)	34 (17.4%)	p = 0.0500^i^

**Table 5:** Comparative study of unstable angina versus myocardial infarction +ve: Positive; -ve: Negative; NSTEMI: Non-ST-segment elevation myocardial infarction; STEMI: ST-segment elevation myocardial infarction.

Total (N = 254)	Marijuana +ve (N = 59)	Two-sample p-value for difference in the proportion	Marijuana -ve (N = 195)	Two-sample p-value for difference in the proportion
Myocardial infarction (NSTEMI and STEMI) (N = 159)	40 (67.7%)	0.4654	119 (61.0%)	0.3628
Unstable angina (N = 95)	19 (32.2%)		76 (38.9%)	

Hospital outcomes

In the marijuana-positive group with ACS, 45.7% were likely to have at least one readmission within one year compared to 35.3% of patients in the marijuana-negative group with ACS, which, however, was not statistically significant (Table 6). The average length of hospital stay during the index admission was 4.29 ± 3.2 days versus 4.21 ± 4.8 days in the marijuana-positive and marijuana-negative groups, respectively (Table 6). There was no death in the marijuana-positive group with ACS, while there were three deaths in the marijuana-negative group with ACS, but the difference was not significant (p = 0.84).

**Table 6:** Comparative study of readmission, the average length of stay, and death in marijuana-positive and marijuana-negative groups ^i^p-values calculated using z-score for comparison of population proportions. ^ii^Wilcoxon rank-sum test. ACS: Acute coronary syndrome; SD: Standard deviation.

Total (N = 254)	Marijuana positive with ACS (N = 59)	Marijuana negative with ACS (N = 195)	p-value
Readmission within one year (N = 96)	27 (45.7%)	69 (35.3%)	0.1498^i^
The average length of hospital stay in days (Mean ± SD)	4.29 ± 3.2	4.21 ± 4.8	1.0000^ii^
Death (N = 3)	0 (0.0%)	3 (1.5%)	0.8459^i^

Ischemia workup

Out of the 59 marijuana-positive patients who had ACS, 10 patients underwent nuclear stress test (NST) and four of them had positive findings for ischemia in NST. Out of those four patients, only two had left heart catheterization done and one patient (50%) had negative findings on the angiogram (Table 7). Out of the 195 marijuana-negative patients who had ACS, 32 patients underwent NST, and 17 of them had findings suggestive of ischemia in NST. Out of those 17 patients, 12 patients had left heart catheterization done. Among those 12 patients, six had negative findings on the coronary angiogram (Table 7).

**Table 7:** Results of NST and cardiac catheterization in marijuana-positive and marijuana-negative patients ^i^p-values calculated using z-score for comparison of the population proportion. ACS: Acute coronary syndrome; NST: Nuclear stress test; cath: Catheterization.

Total (N = 254)	Marijuana positive with ACS (N = 59)	Marijuana negative with ACS (N = 195)	p-value
Number of patients with NST done	10	32	
NST-positive patients	4	17	
NST positive with cath done	2	12	
NST positive and cardiac cath negative	1 (1.6%)	6 (3.1%)	0.4237^i^

## Discussion

Marijuana and smoking

Our study found that marijuana users were more likely to be males of a relatively younger age group and of African American descent. These findings collaborate with the findings of the national in-patient survey [11]. However, none of these differences reached statistical significance in our study. As seen in studies done previously, marijuana users were more likely to be smokers and cocaine users. In this study, the patients who used marijuana and had ACS were less likely to be obese, have dyslipidemia, or have peripheral vascular disease. A fewer number of patients from this cohort have a personal or family history of CAD. However, they were more likely to have hypertension as opposed to findings in an earlier study [10].

Studies have shown that marijuana use significantly decreased the time for the development of angina symptoms compared to smoking tobacco [14,15]. The probable mechanism is an increase in venous carboxyhemoglobin levels, thus increasing myocardial oxygen demand and decreasing the oxygen delivery to the heart [14]. Our study found that marijuana users who suffered from ACS were more likely to have MI compared to unstable angina; however, the difference was not clinically significant (Table 5). Among patients who suffered from ACS, the chances of having STEMI were significantly higher in marijuana users compared to non-users in our study. The high incidence of STEMI in marijuana users is also reflected in various case reports who presented with MI after cannabinoid use [4-8]. Our study defined marijuana use based on urine toxicology findings and not on the history of marijuana use. That is why the time of last marijuana use could not be ascertained to find out whether patients who suffered from ACS had used it within the last 60 minutes of symptom onset as seen in an earlier study [10].

We selected a population of 18-54-year age group to choose the 90th percentile of the marijuana user population. Besides, we also wanted to remove the confounding effects of older age and the traditional risk factors for ischemic heart disease associated with advanced age.

Cardiovascular effects in younger adult

In our study, we have shown that marijuana use independently increased the likelihood of causing ACS in the younger age group of 18-36 years. This matches the findings of a French study in which the mean age of patients with marijuana use who had cardiovascular complications was 34.8 ± 8.8 years. The study demonstrated that 1.8% of all cannabis-related events reported to the French Addictovigilance Network from 2006 to 2010 were cardiovascular. Twenty out of 35 cardiovascular events were due to ACS [16]. Another retrospective study done to look into all hospitalization caused by cannabis use found that cardiovascular disorders constituted 9.5% of all the adverse events causing hospitalization [17].

Mechanism of ACS

The exact mechanism by which marijuana leads to ischemic events remains unknown. It has been proposed that marijuana has multifactorial effects on cardiovascular physiology [18,19]. The major mechanism contributing to acute ischemic stroke by marijuana use in the nationwide in-patient sample was postulated to be due to vasospasm [11]. In this study, one out of two (50%) patients who had positive NST among the marijuana-positive cohort had negative findings on coronary angiography. The positive NST results without significant coronary stenosis could be explained by marijuana-induced vasospasm.

Marijuana-induced vasospasm was demonstrated by means of cardiac magnetic resonance imaging as the mechanism of STEMI in a pediatric case report, in which the symptoms, electrocardiographic changes, and imaging abnormalities reversed after stopping marijuana [20]. There have been case reports of patients presenting with inferior wall STEMI [21] and ventricular tachycardia [22] after marijuana consumption who were found to have slow coronary flow in angiogram in the absence of any coronary stenosis. This possibly points toward the effect of marijuana on coronary microcirculation [18]. Aronow et al. postulated that compared to smoking high nicotine cigarettes, smoking marijuana decreased the time to angina onset by increasing the heart rate, blood pressure, cardiac output, and venous carboxyhemoglobin levels [23]. Besides, in vitro study has demonstrated that human platelets have CB1 and CB2 receptors on the cell membrane, and delta-9-tetrahydrocannabinol (THC) may have pro-coagulatory effects on these receptors [24], which could lead to thrombosis of the coronary artery without any underlying stenosis or coagulation disorders as explained in some case reports [25].

Mittleman et al. demonstrated in their study that 60 minutes following marijuana use, the risks of MI increased by 4.8 times, and then the risk vanishes after one hour [10]. Similarly, studies have concluded that MI can be triggered two hours after an episode of anger [26], and the risks of MI increase one hour following cocaine use by 23.7 times [27]. These studies help to conclude that marijuana causes delta-9-tetrahydrocannabinol-mediated sympathetic stimulation and reduced parasympathetic activity, which peaks in 15 minutes after maximal THC concentration and lasts for three hours [28]. To know the long-term effect of THC on the coronary vasculature, studies with a longer duration of follow-up are required.

Limitations of the study

Our study lacked the data regarding last cannabinoid use, duration, and frequency of marijuana smoked by the study population; so, it is difficult to tell whether the significant difference in the prevalence of ACS seen in the younger population is due to the acute or long-term impact of marijuana use. This study lacked the data regarding concurrent use of other substances such as synthetic marijuana and amphetamine by the study population that has been known to cause ACS [29,30]. Besides, the number of marijuana-positive patients in the 18-36 age group who had ACS was relatively small in our study, so a larger study of ACS patients within the younger patient population would help to corroborate these findings.

## Conclusions

Marijuana use has been on the rise across the US. It has been legalized for recreational use in several states despite inadequate data in regard to its cardiovascular effects and safety. This retrospective study found that in younger patients (age 18-36 years), marijuana use is independently associated with a five-fold higher risk of ACS. More studies with a larger sample size are needed to collaborate on this finding.
